# The clinical follow-up and management of COVID-19 in children and adolescents with an immunocompromised state or a malignancy

**DOI:** 10.55730/1300-0144.5348

**Published:** 2022-01-05

**Authors:** İrem Ceren ERBAŞ, Hatice KARAOĞLU ASRAK, Ayşe ÇAKIL GÜZİN, Şilem ÖZDEM ALATAŞ, Şefika AKYOL, Meral TORUN BAYRAM, Refik Emre ÇEÇEN, Dilek İNCE, Özlem TÜFEKÇİ, Nurşen BELET

**Affiliations:** 1 Department of Pediatric Infectious Disease, Faculty of Medicine, Dokuz Eylül University, İzmir, Turkey; 2 Department of Pediatric Hematology and Oncology, Faculty of Medicine, Dokuz Eylül University, İzmir, Turkey; 3 Department of Pediatric Nephrology, Faculty of Medicine, Dokuz Eylül University, İzmir, Turkey

**Keywords:** Coronavirus, immunosuppression, kidney transplantation, leukemia, pediatrics

## Abstract

**Background/aim:**

It is still not known how an immunosuppressive state affects the response to coronavirus disease 2019 (COVID-19) in children and adolescents. The aim of this study was to evaluate clinical characteristics, outcomes, and follow-up results of COVID-19 in pediatric patients with a history of immunocompromise or malignancy, retrospectively.

**Materials and methods:**

Patients with a diagnosis of COVID-19 who were under 18 years of age and had a history of immunosuppressive chronic disease or under immunosuppressant treatment were included in the study. Patients were applied to our outpatient clinic or consulted to our department in a tertiary center during the first year of the pandemic.

**Results:**

We evaluated 18 patients with a median age of 15.0 (0.6–17.8) years. Twelve patients (66.6%) were tested because of a symptom and the most common symptom was fever (44.4%, n = 8). Ten of the symptomatic patients (55.5% of all cohort) had a mild disease, the remaining two patients (11.1%) with an end-stage malignancy had critical diseases. Twelve patients (66.7%) were managed on an outpatient basis and were followed up at home, while the remaining six (33.3%) required hospitalization. One patient, who had Ewing sarcoma, died during the follow-up in the intensive care unit, and others were recovered without any morbidities. Lymphocyte (LYM) counts were significantly lower, C-reactive protein (CRP), and ferritin levels were higher in the individuals that needed hospitalization (p = 0.039, 0.027, and 0.039, respectively).

**Conclusion:**

Immunocompromised children and adolescents with COVID-19 should be monitored closely, especially those with an end-stage malignancy, low LYM count, or high CRP and ferritin levels.

## 1. Introduction

In 2019, newly identified cases of severe acute respiratory syndrome coronavirus-2 (SARS-CoV-2) were reported and spread rapidly all over the world from the Hubei province of China [1]. On 11^th^ March 2020, the World Health Organization (WHO) declared the coronavirus disease 2019 (COVID-19) as a pandemic. By March 2021, there have been more than 125 million confirmed cases around the globe, including more than 2.7 million deaths, reported by WHO^1^.

Although children are known to be affected by COVID-19 to varying degrees of severity, severe and mortal consequences are rare in pediatric patients compared to adults [2–6]. This may be related to the fact that children have fewer comorbidities, have lower angiotensin-converting enzyme 2 (ACE2) receptor expression, and produce a different inflammatory response involving a higher number of B and T-regulatory cells [7,8]. Even though the pathophysiology of COVID-19 has not yet been fully understood, critically ill patients have high levels of cytokines, particularly interleukin-6 (IL-6), suggesting that hyperinflammation may contribute to morbidity and mortality in this disease [7,9].

It is still not known how an immunosuppressive state affects the response to COVID-19 in children and adolescents. Limited data suggest that immunocompromised children and adults mostly have a mild disease compared to the general population [10]. Conversely, there are also publications showing that adult patients with cancer had worse outcomes than those without cancer [11–16]. To date, several reports demonstrated the impact of this pandemic on the immunocompromised population and most of them included adults. Clinical outcomes, the severity of the disease, and the effectiveness of treatment methods are still unclear in immunocompromised children and adolescents diagnosed with COVID-19 [17].

In this study, we aimed to evaluate clinical characteristics, outcomes, and follow-up results of COVID-19 in the pediatric population with a history of immunocompromise or malignancy.

## 2. Materials and methods

Patients with a diagnosis of COVID-19 who were under 18 years of age and had a history of immunosuppressive chronic disease or under immunosuppressant treatment were included in the study. Patients were applied to our outpatient clinic or consulted to our department in a tertiary center during the first year of the pandemic in Turkey (March 2020–March 2021). Relevant data were collected retrospectively from the clinical records of the patients.

A COVID-19 reverse transcriptase-polymerase chain reaction (RT-PCR) test was performed from the nasopharyngeal swab taken before hospitalization, surgical procedures, chemotherapy, and radiotherapy, or in case of any clinical symptoms associated with this disease. A confirmed case of COVID-19 was defined as a positive result by RT-PCR on a nasopharyngeal swab specimen.

Demographic characteristics, contact histories, comorbidities, clinical symptoms, laboratory test results, radiological findings, and severity of the disease for all patients were noted at the first admission. Laboratory examinations, including complete blood count, inflammatory or infection-related biomarkers, cardiac, renal, liver, and coagulation function tests were obtained at the initial diagnosis time and during the follow-up period. Blood culture from febrile patients and a urine culture from those with symptoms related to urinary tract infections were obtained. Imaging methods such as chest X-rays and computerized tomography (CT) were decided on an individual basis. Details of applied treatment methods, follow-up period, and clinical outcomes (need of an intensive care unit, morbidities, mortality) were also noted.

An asymptomatic disease was defined as COVID-19 PCR positive in the patient who had no symptoms. A mild disease was defined for patients with SpO_2_ > 94% and who did not require supplemental oxygen. Severe disease was defined for those with SpO_2_ ≤ 94% at room air or who received supplemental oxygen. A critical illness was defined as an end-organ dysfunction, acute respiratory distress syndrome (ARDS), need for mechanical ventilation, or extracorporeal mechanical oxygenation [18].

Acute respiratory distress syndrome was classified using the Berlin criteria (PaO_2_/FiO_2_ ratio 200–300 mild, 100–200 moderate, and <100 severe ARDS) [19]. Sepsis, severe sepsis, and septic shock states were described according to the pediatric sepsis guidelines [20]. Febrile neutropenia was defined in individuals with hematological malignancies who showed an absolute neutrophil count (NEU) of less than 500/mm^3^ or between 500–1000/mm^3^ and an expected NEU to decrease below 500/mm^3^ within 24–48 h, and who had a fever as a single temperature of ≥38.3 °C (101 °F) or ≥38.0 °C (100.4 °F) sustained over an hour [18]. A diagnosis of hemophagocytic lymphohistiocytosis (HLH) was defined according to the HLH-2004 diagnostic criteria [21].

Normal ranges of laboratory tests were admitted as follows; white blood cell count (WBC) 4000–10,300/mm^3^, NEU 2100–6100/mm^3^, lymphocyte count (LYM) 1300–3500/mm^3^, monocyte count (MONO) 300–900/mm^3^, hemoglobin (HGB) 13.5–17.5 gr/dL, platelet count (PLT) 156,000–373,000/mm^3^, C-reactive protein (CRP) 0.2–5 mg/L, procalcitonin (PCT) 0–0.05 ng/mL, aspartate aminotransferase (AST) 15–60 U/L, alanine transaminase (ALT) 13–45 U/L, urea 5–18 mg/dL, creatinine 0.2–0.4 mg/dL, albumin 3.5–5.2 g/dL, lactate dehydrogenase (LDH) 180–360 U/L, D-dimer: 0–0.55 ug/mL, fibrinogen 1.8–3.5 g/L, high sensitivity-troponin (Hs-Troponin) I 8.4–18.3 ng/L, and ferritin 11–306 ng/mL.

This study was approved by the local ethics committee (ethics approval number: 2021/08-40) and the Ministry of Health and performed according to the principles of the Declaration of Helsinki. An informed written consent form was not obtained due to the retrospective nature of the study.

### 2.1. Statistical analysis

All statistical analyses were performed using the SPSS application for Windows version 24.0 (IBM Co., Armonk, NY, USA). Clinical data were presented as counts and percentages, or medians with the respected minimum-maximum values. Comparisons of medians were performed using the Mann-Whitney U test. The Pearson chi-square test, or Fisher Exact test if any expected cell size numbered <5, was used for the comparison of categorical data. A p-value of <0.05 was considered statistically significant.

## 3. Results

We included 18 patients with COVID-19 diagnosis, who had an underlying chronic disease that causes immunosuppression or received immunosuppressive therapy. The median age of the patients was 15.0 years (0.6–17.8 years) and 11 (61.1%) were male. Four of the patients (22.2%) had hematological malignancies, 11 (61.1%) had solid tumors, one (5.6%) had a history of recent renal transplantation and, two (11.2%) were using immunosuppressant agents for focal segmental glomerulosclerosis and lupus nephritis, separately (Table 1). Fifteen patients (83.3%) were diagnosed in the outpatient clinic. The remaining three patients (16.7%), who were already hospitalized for reasons related to their primary diseases, were diagnosed after developing any symptoms associated with COVID-19.

Six cases (33.3%) had a history of close contact with confirmed COVID-19 cases. Six asymptomatic patients (33.3%) were tested for COVID-19 before hospitalization for any surgical or treatment procedures. Twelve patients (66.7%) were tested because of a symptom, beginning a median of 1.5 (1–6) days before testing. The most common symptoms were fever (44.4%, n = 8), cough (16.7%, n = 3), sore throat (16.7%, n = 3), nasal congestion (16.7%, n = 3), loss of smell (6.6%, n = 1), and diarrhea (6.6%, n = 1), respectively. Two patients (Case 1 and 3) had febrile neutropenia at the time of diagnosis. None of the patients had a positive blood culture. One of the patients (Case 2) demonstrated infiltrations on the chest radiograph related to the metastasis of primary disease, without any sign of pneumonia on the physical examination (Table 2).

Treatment characteristics and follow-up results of the immunocompromised children with COVID-19 are shown in Table 2. In our patient cohort, 12 (66.7%) were managed on an outpatient basis and were followed up at home, while the remaining six (33.3%) required hospitalization, three of whom were already hospitalized due to their primary diseases. Reasons for hospitalization in other patients were found as follows; a diagnosis of critical disease in one (Case 3), the detection of Pseudomonas aeruginosa in the urine culture in one (Case 12), and caregivers were unable to provide adequate care in the remaining one (Case 4) (Table 2). The median length of hospital stay due to COVID-19 was 7.5 days (5–15 days). Two patients (11.1%) with an end-stage malignancy had critical diseases and were followed up in the intensive care unit.

Seventeen of the patients (94.4%) recovered from COVID-19 without any morbidities. One patient (Case 10) died after developing HLH and ARDS, on the 15^th^ day of PCR positivity. The cytokine storm could not be prevented despite additional treatment methods. On the follow-up of other patients who recovered from COVID-19, two individuals died due to their end-stage malignancies, after one and four months of discharge. A 17-year-old female patient (Case 17) who received intensive immunosuppressant treatment protocols was diagnosed as COVID-19 only 22 days after kidney transplantation. Although she was asymptomatic on the initial examination, she complained of sore throat and developed lymphopenia seven days after the diagnosis. Therefore, doses of immunosuppressive agents were decreased, but she did not require hospitalization and followed-up without any complications or graft rejection.

When we compared laboratory results, LYM counts were lower, CRP and ferritin levels were higher in the individuals who needed hospitalization (p = 0.039, 0.027, and 0.039, respectively). Six of the patients were received chemotherapies (40% in those with a malignancy) in the last 14 days before the diagnosis of COVID-19 and also two patients were under immunosuppressant treatments at the time of diagnosis. A history of immunosuppressant treatment on the last 14 days before the diagnosis did not differ among groups who were hospitalized or followed up at home (p = 1.000) (Table 3).

## 4. Discussion

A critical disease due to COVID-19 is rare in children, but its course in immunocompromised children has not been clearly known [4,5,10]. On the other hand, it has been reported that adult cancer patients with COVID-19, especially those with a metastatic malignancy, have a higher risk of severe disease and death than those without cancer. Also, it has been recommended to avoid immunosuppressant treatments in these patients [12–14,16]. Although COVID-19 infection is mostly asymptomatic in the general pediatric population, the rate of having an asymptomatic disease in immunosuppressed children and adolescents has been reported to range between 33%–84% [11,22–24]. The most common symptoms in these patients were found as fever and cough in the previous studies [11,23,24]. In this study, 66.6% of the patients were symptomatic and the most common symptoms were fever and cough. Our results were similar to the literature data including the reports from our country, however, varying frequencies in different studies may be due to the limitation of the number of patients. In addition, the increased rate of symptoms in immunocompromised children rather than the general population may be related to accompanying problems and impaired immune response. However, underlying pathophysiologic mechanisms for this have not been elucidated yet.

In recent studies, most pediatric cancer patients with COVID-19 were reported to have a relatively mild illness. It was claimed that hospitalized cancer patients with COVID-19 were often admitted because of problems with their primary diseases rather than complications of COVID-19 infection [11,23–26]. Most of these patients were applied to the outpatient clinics and hospitalization rates due to COVID-19 were ranged up to 74% in several studies [23,24,27]. Similar to the previous reports, most of our patients had mild disease, whereas 33.3% of this cohort were followed up in the hospital. Although it is known that lymphopenia and elevation in CRP and ferritin levels are common laboratory features in COVID-19 infection, some studies in the pediatric population showed no difference in these parameters according to the severity of the disease [3,22]. Herein, we demonstrated that LYM counts were lower, CRP and ferritin levels were higher in those who were hospitalized, pointing out that these parameters should be carefully evaluated in immunocompromised children and adolescents.

The first COVID-19 case of an immunocompromised child was an 8-year-old boy with a diagnosis of acute lymphoblastic leukemia, who followed up with mechanical ventilation support for three weeks due to respiratory failure and died due to HLH [28]. In the literature, rates of only severe and critical illness were found as 2%–5% and 0.6%–0.7% of the general pediatric population with COVID-19, respectively [5,29]. A need for respiratory support and intensive care is reported between 0%–32% in immunocompromised pediatric patients, including the results of studies from our country [11,23,24,30]. In the Global COVID-19 in Childhood Cancer Registry, a severe/critical disease frequency was found as 18.4% and death was reported in 3.4% of children with malignancy [31]. Our findings were all in accordance with the literature data, showing that severe and critical COVID-19 infections were more common in these groups compared to the general pediatric population. However, the clinical progress of COVID-19 was less severe in pediatric cancer patients compared to adults. This may be related to the low affinity of this virus to children. We demonstrated that 11.1% of our patients were followed up in the intensive care unit, all of whom had a concomitant end-stage malignancy with extensive metastases. COVID-19 may cause severe disease in those with end-stage cancer, therefore, these patients should be protected against this infection carefully and should be monitored closely after a diagnosis of COVID-19.

Zhang et al. [12], found that adult cancer patients who received antitumor treatments in the previous 14 days had a significantly higher risk of serious events in COVID-19 infection. In a study with pediatric cancer patients, 60% of them have received chemotherapy within 15 days before COVID-19 diagnosis [27]. Some authors claimed that COVID-19 may lead to adverse outcomes in patients with malignancy, particularly in those with febrile neutropenia [25,26]. In this study, 40% of the patients with cancer had received chemotherapy in the last 14 days before the diagnosis of COVID-19 and two of them were admitted to the intensive care unit, one died. Our three patients, who received immunosuppressive agents other than chemotherapy, had a mild clinical course. However, whether or not the treatment was given in the last 14 days was not different among the hospitalized and outpatient groups. These findings suggested that cancer or chemotherapy may be responsible for the poor prognosis in COVID-19, rather than other underlying diseases that cause immunosuppression. Although febrile neutropenia was thought to be a risk factor for a severe COVID-19 infection, we could not observe this in our patients. Despite we did not have enough patients with febrile neutropenia to come to a definite conclusion, alternative immunological pathways might be involved in response to this virus in these patients.

Immunosuppressive agents used in the management of kidney transplant recipients and glomerular diseases cause lymphopenia and impaired lymphocyte functions. The use of high doses of immunosuppressants, especially in the first three months after the transplantation, concerns the development of severe COVID-19 disease in this patient group [9]. In literature, COVID-19 infections were reported mostly in adulthood in renal transplant patients, and information for the pediatric age group is very limited. In adults, mild, severe, and critically ill patients were all seen, and also deaths were reported. These transplant patients with a severe or critical COVID-19 disease had comorbidities such as hypertension and diabetes mellitus [32–35]. In addition, Angeletti et al. [36], suggested that chronic immunosuppression might not be associated with an increased risk of COIVD-19 in young kidney transplant recipients. Several studies showed that the clinical outcomes of COVID-19 in kidney transplant recipients were not significantly different than nontransplant individuals [33,36]. Besides, in a recent publication, it was reported that pediatric renal transplant recipients had a mild disease of COVID-19 [37]. In this study, we presented one patient with recent renal transplantation, one patient with focal segmental glomerulosclerosis, and one patient with lupus nephritis, who was under immunosuppressive therapy. These patients were followed up with a mild clinical condition or asymptomatic for COVID-19. To the best of our knowledge, we presented the first pediatric case to become infected at such an early stage after the transplantation. This result made us think that immunosuppressive treatment regimens used in renal transplantation or nephritis may not alter the immune response to COVID-19 in children and adolescents.

Gampel et al. [23], reported that only 16% of their patient cohort received treatment for COVID-19 and most of them were given hydroxychloroquine. In another report, most patients (73%) received hydroxychloroquine and some of them were given in different combinations with remdesivir or lopinavir-ritonavir. Also, they found pathological findings in chest radiographs in 57% of patients [27]. In this study, 27.7% of our patients received hydroxychloroquine or favipiravir treatments. However, there were no signs of any infiltrations due to infection in chest radiographs among available data. Since there is a lack of evidence-based guidelines for treatment approaches to COVID-19 in immunocompromised children and adolescents, it may be appropriate to make patient-based decisions. Also, a routine imaging method for lungs seems to be unnecessary, especially in asymptomatic pediatric patients. Although we started anticoagulant treatments in some individuals, there were no thrombotic complications that occurred in either treated- or untreated groups. Therefore, prospective randomized-controlled trials should be performed to create diagnostic and therapeutic guidelines for these specialized patient groups.

Herein, we presented one of the largest cohorts from a single center until today, in children and adolescents with malignancy or an immunosuppressive state. However, this study has some limitations. First of all, it was a retrospective study and our cohort included a small number of patients. Nevertheless, this was a consequence of the rarity of COVID-19 in immunocompromised children and adolescents. Although the infection is commonly seen around the world, it less affects the pediatric age group. Also, pediatric patients with a concomitant immunosuppression status are usually well protected by their parents against infections. It may be difficult to interpret the data and come to a definite conclusion with a such limited patient group, but all novel information about COVID-19 in special patient groups is valuable for researchers. More prospective and molecular studies with larger sample sizes are needed in this subject to elucidate the molecular and pathophysiological mechanisms, as well as long-term follow-up results in these patients.

In conclusion, COVID-19 was found to be milder in children and adolescents who had malignancy or under immunosuppressive treatment, than in adults. However, when compared with the studies in the general pediatric population, the presence of a severe or critical illness was higher. Those with an end-stage malignancy, low LYM count, or high CRP and ferritin levels should be monitored closely after the diagnosis of COVID-19.

## Figures and Tables

**Table 1:** Demographic, clinical characteristics, and laboratory results of the immunocompromised children with COVID-19.

Case	Sex	Age (Years)	Underlying disease	Metastasis	Immuno-suppressive treatment	Last day of the treatment (before the diagnosis)	Clinic that the patient was diagnosed	The severity of the disease	Coinfections	WBC (/mm^3^)	NEU (/mm3)	LYM (/mm3)	CRP (mg/L)	PCT (ng/mL)	Ferritin (ng/mL)	D’dimer (ug/mL)	Hs-Trop (ng/L)
**1**	F	11.4	AML	-	CT	10 days	Inpatient	Mild disease	-	500	200	300	61	0.21	782	0.4	6.9
**2**	M	13.9	Ring-cell gastric cancer	+	-	N/A	Inpatient	Mild disease	-	6600	5600	700	113	0.11	957	26.1	28.4
**3**	F	15.4	Orbital RMS	+	CT	6 days	Inpatient	Critical disease	-	900	100	600	570	11.19	4065	2.8	13.6
**4**	M	14.1	Germ-cell tumor	-	-	N/A	Inpatient	Asymptomatic	-	7700	4700	2000	0.4	0.02	18	0.2	5.6
**5**	M	16.5	Desmoid tumor	-	CT	10 days	Outpatient	Mild disease	UTI	10300	7500	2000	11.2	0.09	10	1.1	N/A
**6**	F	4.5	ALL	-	CT	33 days	Outpatient	Asymptomatic	-	4800	3100	1000	0.2	0.01	458	0.4	50
**7**	M	8.5	Malignant melanoma	-	CT	360 days	Outpatient	Asymptomatic	-	4400	1300	2200	1.7	N/A	130	0.5	5.6
**8**	M	0.6	Neuroblastoma	-	Operated	N/A	Outpatient	Mild disease	UTI	20400	11100	5400	77.8	0.83	201	1.2	5.8
**9**	M	15	Osteosarcoma	-	CT	5 days	Outpatient	Mild disease	-	900	0	800	36.5	0.51	865	0.5	29.8
**10**	M	16.3	Ewing sarcoma	+	CT	10 days	Inpatient	Critical disease	-	1700	1200	500	256.7	2.74	10900	9.2	14
**11**	F	14.9	ALL	-	CT	24 days	Outpatient	Asymptomatic	-	5700	3000	2300	7.3	0.11	N/A	0.3	6.9
**12**	M	8.2	ALL	-	CT	150 days	Inpatient	Mild disease	UTI	4700	4000	700	78	0.13	3981	3.4	7.1
**13**	M	15	Burkitt lymphoma	+	CT	13 days	Outpatient	Asymptomatic	-	N/A	N/A	N/A	N/A	N/A	N/A	N/A	N/A
**14**	M	3.5	Wilms tumor	-	CT	18 days	Outpatient	Mild disease	-	3000	500	1700	7.6	0.11	164	0.5	54
**15**	F	17	Ewing sarcoma	-	CT	20 days	Outpatient	Mild disease	-	2100	1200	600	2.4	0.04	N/A	0.3	5.6
**16**	F	17.5	Lupus nephritis	N/A	Mycophenolate mofetil	Under treatment	Outpatient	Mild disease	-	4200	2800	900	0.6	0.1	21.6	0.7	5.6
**17**	F	17.8	Kidney transplantation	N/A	Mycophenolate mofetil, tacrolimus, glucocorticoid	Under treatment	Outpatient	Asymptomatic	-	8900	8100	600	9.2	0.04	N/A	0.5	5.6
**18**	M	17.3	FSGS	N/A	Rituximab	7 days	Outpatient	Mild disease	-	5900	4300	1200	1.4	0.03	90.7	0.17	5.6

ALL: Acute lymphoblastic leukemia, AML: Acute myeloid leukemia, CRP: C-reactive protein, CT: Chemotherapy, F: Female, FSGS: Focal segmental glomerulosclerosis, Hs-Trop: High-sensitivity troponin, LYM: Lymphocyte count, M: Male, N/A: Not available, NEU: Neutrophil count, PCT: Procalcitonin, RMS: Rhabdomyosarcoma, UTI: Urinary tract infection, WBC: Leukocyte count.

**Table 2:** Treatment characteristics and follow-up results of the immunocompromised children with COVID-19.

Case	Urinary culture	Chest X-ray	Thoracic CT	ECHO	Clinical follow-up	Antiviral therapy	Antibiotic therapy	Anticoagulant therapy	Additional treatments	Duration of follow-up in ICU (days)	Duration of hospitalization (days)	Complications	Death
**1**	-	Normal	-	-	Hospitalized	HC	-	-	-	-	5	-	-
**2**	-	Infiltration	Metastatic lesions	Mitral insufficiency	Hospitalized	HC	+	-	-	-	5	-	-
**3**	-	Normal	Normal	Normal	Hospitalized	-	+	+	O_2_ support, inotrope	5	11	Shock state	-
**4**	-	Normal	-	-	Hospitalized	HC	-	-	-	-	5	-	-
**5**	*Enterococcus spp*.	Normal	-	-	At home	HC	+	-	-	-	0	-	-
**6**	-	Normal	-	-	At home	-	-	-	-	-	0	-	-
**7**	-	Normal	-	-	At home	-	-	-	-	-	0	-	-
**8**	*Escherichia coli*	N/A	-	-	At home	-	+	-	-	-	0	-	-
**9**	-	Normal	-	Normal	At home	-	-	-	-	-	0	-	-
**10**	-	Normal	-	-	Hospitalized	Favipiravir	+	+	Mechanical ventilation, corticosteroid, inotrope, plasma exchange, anakinra	14	15	ARDS, HLH	+
**11**	-	Normal	-	-	At home	-	-	-	-	-	0	-	-
**12**	*Pseudomonas aeruginosa*	Normal	-	-	Hospitalized	-	+	+	IVIG	-	10	-	-
**13**	-	N/A	-	-	At home	-	-	-	-	-	0	-	-
**14**	-	Normal	-	Normal	At home	-	-	-	-	-	0	-	-
**15**	-	Normal	-	-	At home	-	-	-	-	-	0	-	-
**16**	-	Normal	-	-	At home	-	+	-	-	-	0	-	-
**17**	-	Normal	-	-	At home	-	-	+	-	-	0	-	-
**18**	-	Normal	-	-	At home	-	-	-	-	-	0	-	-

ARDS: Acute respiratory distress syndrome, CT: Computerized tomography, ECHO: Echocardiography, HC: Hydroxychloroquine, HLH: Hemophagocytic lymphohistiocytosis, ICU: Intensive care unit, IVIG: Intravenous immunoglobulin, N/A: Not available.

**Table 3:** Comparison of laboratory results and immunosuppressant treatment status on the last 14 days among the patients that were hospitalized or followed up at home.

	At home (n = 12)	Hospitalized (n = 6)	p
**White blood cells (/mm** **^3^** **) (n = 17)**	4500 (2475–9525)	3200 (800–6875)	0.291^a^
**Neutrophile (/mm** **^3^** **) (n = 17)**	2950 (375–6000)	2600 (175–4925)	0.580^a^
**Lymphocyte (/mm** **^3^** **) (n = 17)**	1100 (875–2625)	650 (450–1025)	**0.039** a
**CRP (mg/L) (n = 17)**	4.5 (0.5–46.8)	95.5 (45.9–335.0)	**0.027** a
**Procalcitonin (ng/mL) (n = 15)**	0.11 (0.03–0.59)	0.17 (0.09–4.85)	0.127^a^
**Ferritin (ng/mL) (n = 14)**	182.5 (73.4–559.8)	2469 (591.0–5773.8)	**0.039** a
**D’dimer (ug/mL) (n = 17)**	0.5 (0.3–0.8)	3.1 (0.4–13.4)	0.117^a^
**Hs-Troponin (ng/L) (n = 16)**	17.8 (5.6–51.0)	10.4 (6.6–17.6)	0.492^a^
**Patients who received chemotherapy or immunosuppressant agent on the last 14 days [n (%)]**	6 (50.0)	3 (50.0)	1.000^b^

CRP: C-reactive protein, Hs-Troponin: High-sensitive troponin. Data were given as median (25–75p).

a Mann-Whitney U test,

b Fisher Exact test, p < 0.05.

## Footnotes

1 World Health Organization (2021). Coronavirus Disease (COVID-19) Dashboard [online]. Website https://covid19.who.int/ [accessed 30 March 2021].
